# Molecular endotyping in systemic lupus erythematosus: an integrative framework toward precision therapy

**DOI:** 10.3389/fimmu.2026.1959612

**Published:** 2026-10-19

**Authors:** Yuya Terajima, Yoshinori Matsumoto, Shoichi Nawachi, Yosuke Asano, Keigo Hayashi, Yoshia Miyawaki, Haruki Watanabe, Eri Katsuyama, Takayuki Katsuyama, Jun Wada

**Affiliations:** 1 Department of Nephrology, Rheumatology, Endocrinology and Metabolism, Okayama University Faculty of Medicine, Dentistry and Pharmaceutical Sciences, Okayama, Japan; 2 Department of Medical Laboratory Science, Okayama University Faculty of Health Sciences, Okayama, Japan

**Keywords:** B cells, molecular endotypes, nucleic acid sensing, precision medicine, single-cell RNA sequencing, spatial transcriptomics, systemic lupus erythematosus, type I interferon

## Abstract

Systemic lupus erythematosus (SLE) is a clinically defined syndrome arising from heterogeneous and dynamically interacting molecular programs. Recent transcriptomic, single-cell, spatial, and functional studies have identified recurrent molecular states; however, published three-, five-, six-, and eight-group taxonomies are not directly interchangeable. In this review, the published evidence was synthesized within a pragmatic framework of four overlapping candidate programs: interferon/interferon-stimulated gene (IFN/ISG), B cell/plasmablast, neutrophil/neutrophil extracellular trap (NET), and an emerging immunometabolic program. Complement/immune-complex activation is considered a cross-cutting effector axis rather than a mutually exclusive endotype. The molecular endotype is defined operationally, and a distinction is made between pathway signatures, candidate endotypes, and clinically actionable endotypes. In this review, activity biomarkers, prognostic biomarkers, pharmacodynamic biomarkers, and predictive biomarkers are distinguished; the transcriptomic ISG-high states are distinguished from directly measurable type I IFN bioactivity; and the contributions of short-lived plasmablasts and long-lived plasma cells to autoantibody production are also examined. It should be emphasized that endotype assignment is time- and compartment-dependent and that blood signatures may not fully capture organ-specific disease, particularly lupus nephritis. Molecular endotyping in SLE should therefore be viewed as a research framework toward precision therapy rather than as a current treatment algorithm. Prospective biomarker-enriched trials and longitudinal multi-compartment profiling will be required to establish clinically actionable endotypes.

## 1 Introduction

Systemic lupus erythematosus (SLE) is a complex systemic autoimmune disease that causes inflammation and damage to various organs, such as the skin, kidneys, joints, hematologic systems, and central nervous systems (1). Some patients experience mild diseases, while others develop severe, life-threatening complications, such as lupus nephritis (LN), neuropsychiatric SLE (NPSLE), and cardiovascular involvement. The heterogeneity in disease manifestations, progression, and response to treatment is attributable to multiple factors, including the genetic background, immune mechanisms, and age of onset. In epidemiology, the global population affected by SLE was estimated to be 3.41 million, with women estimated to be 3.04 million (2). The incidence of SLE varies by country and race; the incidence among Black individuals is three times that of White individuals (age-adjusted incidence rates of 10.7 versus 3.3 per 100,000 person-years) (3). Advances in transcriptome analysis methods have revealed the importance of both type I interferon (IFN)-dependent and -independent gene signatures in the pathogenesis of SLE (4). This heterogeneity is associated with the scarcity of universal biomarkers capable of predicting SLE recurrence or treatment response, as well as the difficulties encountered in conducting clinical trials for the development of therapeutics.

SLE is characterized by an increased positivity rate of autoantibodies, such as anti-double-stranded deoxyribonucleic acid (dsDNA) and anti-Smith antibodies. It is believed that autoantibodies secreted by plasmablasts and long-lived plasma cells activate the immune system by cross-linking the Fc region of immunoglobulin G (IgG), either individually or as a part of immune complexes, thereby initiating the activation of the complement cascade. In recent years, mouse models and patients with severe SLE have achieved remission with anti-CD19 chimeric antigen receptor (CAR) T cell therapy, suggesting that B cell depletion supports the central pathogenic role of autoreactive B cell programs and autoantibody networks (5–7). Clinically, SLE is characterized not only by diverse autoantibodies but also by fluctuating type I IFN activity, immune complex-driven tissue injury, and marked molecular heterogeneity across patients. Recent advances in single-cell ribonucleic acid sequencing (scRNA-seq), spatial transcriptomics, and high-resolution flow cytometry have redefined the pathogenesis of SLE. Large whole-blood transcriptomic and single-cell studies have identified candidate molecular clusters associated with disease activity, serological features, organ involvement, and, in some studies, exploratory treatment-response patterns (8–11). **Table 1** summarizes these classifications. However, the number and composition of these clusters vary according to cohort characteristics, assay platform, sample compartment, disease activity, treatment exposure, and analytical method. None of the published classifications has yet been established as a universal treatment-selection taxonomy for routine clinical practice.

**Table 1:** The list of published SLE molecular taxonomies.

Author	Garantziotis P, et al. (2022)	Hubbard E, et al. (2023)	Nehar-Belaid D, et al. (2020)	Cui M, et al. (2021)
Research Methods	Cross-sectional endotyping and in silico, hypothesis-generating drug prediction	Cross-sectional meta-stratification and *post hoc* exploratory trial response analysis	Cross-sectional single-cell profiling and observational treatment association	Cross-sectional subtype classification and retrospective exploratory response association
Cohort	120 adult SLE patients and 58 healthy controls	3,166 SLE patients across 17 datasets	Cohort 1: 33 pediatric SLE patients and 11 healthy controls Cohort 2: 8 adult SLE patients and 6 healthy controls	Cohort 1: 158 pediatric SLE patients and 72 healthy controls Cohort 2: 62 adult SLE patients and 20 healthy controls
Samples	Whole blood cells	Whole blood cells	PBMCs	Whole blood cells
Platform	Bulk RNA-seq	Microarray and bulk RNA-seq	Single-cell RNA-seq	Microarray
Number of groups	5 molecular endotypes (G1 through G5)	8 molecular endotypes (A through H)	6 molecular endotypes (SCG1 through SCG6)	3 molecular endotypes (mixed, IFN-high, NE-high)
Dominant molecular features	G1 (hemostasis): platelet activation and hemostasis G2 (autophagy): macroautophagy, neutrophil activation, and TLR cascades G3 (metabolism): mRNA splicing/surveillance and mitochondrial dysfunction/metabolic disturbances G4 (neutrophil): neutrophil activation and degranulation G5 (B cell): B cell and plasmablast-mediated responses	A: least abnormal and minimal gene expression dysregulation B: predominantly driven by T cell signatures C: high myeloid cell signature D: predominantly IFN and TNF signature E: prominently features cell cycle, IFN, plasma cell, and myeloid signatures F: not only prominent T cell signature but also myeloid signature G: prominently features myeloid cell, TNF, IFN, and inflammasome signature H: most abnormal and high enrichment of monocytes, neutrophils, TNF, granulocytes, LDGs, and anti-inflammatory signatures	SCG1: prominent expansion of ISG^high^ subclusters across monocytes, DN2 B cells, plasma cells, T cells, and NK cells SCG2: Specific expansion of erythrocyte and megakaryocyte subclusters SCG3: expansion of ISG^high^ dendritic cell and monocyte subclusters SCG4: combined expansion of ISG^high^ dendritic cell subclusters and non-ISG^high^ B cell subclusters SCG5: enrichment of ISG^low^ subclusters across T cells, B cells, NK cells, pDCs, and cDCs SCG6: dominance of ISG^low^ subclusters similar to healthy controls with minimal transcriptomic perturbation	mixed: simultaneous dysregulation of type I IFN, viral infection pathways, bacterial/fungal infection, and NETosis pathways IFN-high: driven predominantly by viral infection response and overactivation of the type I IFN signaling pathway NE-high: driven predominantly by bacterial/fungal infection responses and neutrophil elastase/NET formation
Clinical associations	G1: high proportion of male patients and DMARD use is common G2: mild-to-moderate SLE, and high rates of photosensitivity and malar rash G3: high prevalence of mucocutaneous and musculoskeletal manifestations, moderate disease activity, and history of NPSLE G4: active LN, and highest clinical and serologic activity G5: high frequency of constitutional symptoms, and trend toward higher prevalence of hematologic and neurologic manifestations	A: the lowest SLEDAI, the lowest anti-dsDNA antibody titers, and the lowest risk of severe flares E: the most disease activity,	SCG1: the highest SLEDAI SCG5 and SCG6: low SLEDAI and like healthy	mixed: the highest SLEDAI and classified more frequently in adult patients and male patients IFN-high: the intermediate SLEDAI and classified more frequently in pediatric patients and female patients NE-high: the lowest SLEDAI
Treatment-response evidence	Predicting effective drugs using transcriptome databases	G/H: depleted due to the treatment with mycophenolate mofetil or methotrexate in combination with glucocorticoid	The association with treatment response has not been explicitly stated	IFN-high: hydroxychloroquine showed the greatest improvement mixed and NE-high: glucocorticoids showed a high improvement effect
Major limitations	Most patients were receiving immunosuppressive agents, and because this is a cross-sectional analysis, it was not possible to assess changes in status over time or disease relapse	Predominantly adult female patients and excluded patients with active LN or NPSLE in this cohort, and most patients were receiving immunosuppressive agents	The reduced survival rate of plasma cells due to cryopreservation, the inability to identify neutrophils due to technical limitations, and most patients were receiving immunosuppressive agents	Reliant on public GEO datasets, excluded 4 patients with stable SLEDAI across visit, and most patients received combination therapy such as glucocorticoid, hydroxychloroquine, and immunosuppressive agents
Reference	(8)	(9)	(10)	(11)

cDC, conventional dendritic cell; DMARD, disease-modifying antirheumatic drugs; DN2, double-negative 2; dsDNA, double-stranded deoxyribonucleic acid; GEO, gene expression omnibus; IFN, interferon; ISG, interferon-stimulated genes; LDG, low-density granulocytes; LN, lupus nephritis; mRNA, messenger ribonucleic acid; NE, neutrophil; NET, neutrophil extracellular trap; NK, natural killer; NPSLE, neuropsychiatric systemic lupus erythematosus; PBMC, peripheral blood mononuclear cell; pDC, plasmacytoid dendritic cell; RNA-seq, ribonucleic acid sequencing; SLE, systemic lupus erythematosus; SLEDAI, systemic lupus erythematosus disease activity index; TLR, Toll-like receptor; TNF, tumor necrosis factor.

Conceptually, these findings support a shift from viewing SLE as one disease driven by a single dominant mechanism toward viewing it as a clinically defined syndrome generated by overlapping and dynamically changing molecular programs. This review adopts a pragmatic translational framework rather than proposing a new definitive taxonomy. The framework is intended to connect recurrent molecular programs with measurable biomarkers, clinical phenotypes, therapeutic vulnerabilities, and the strength of supporting evidence. The integrated architecture of the pathogenesis of SLE is illustrated in **Figure 1**.

**Figure 1:**
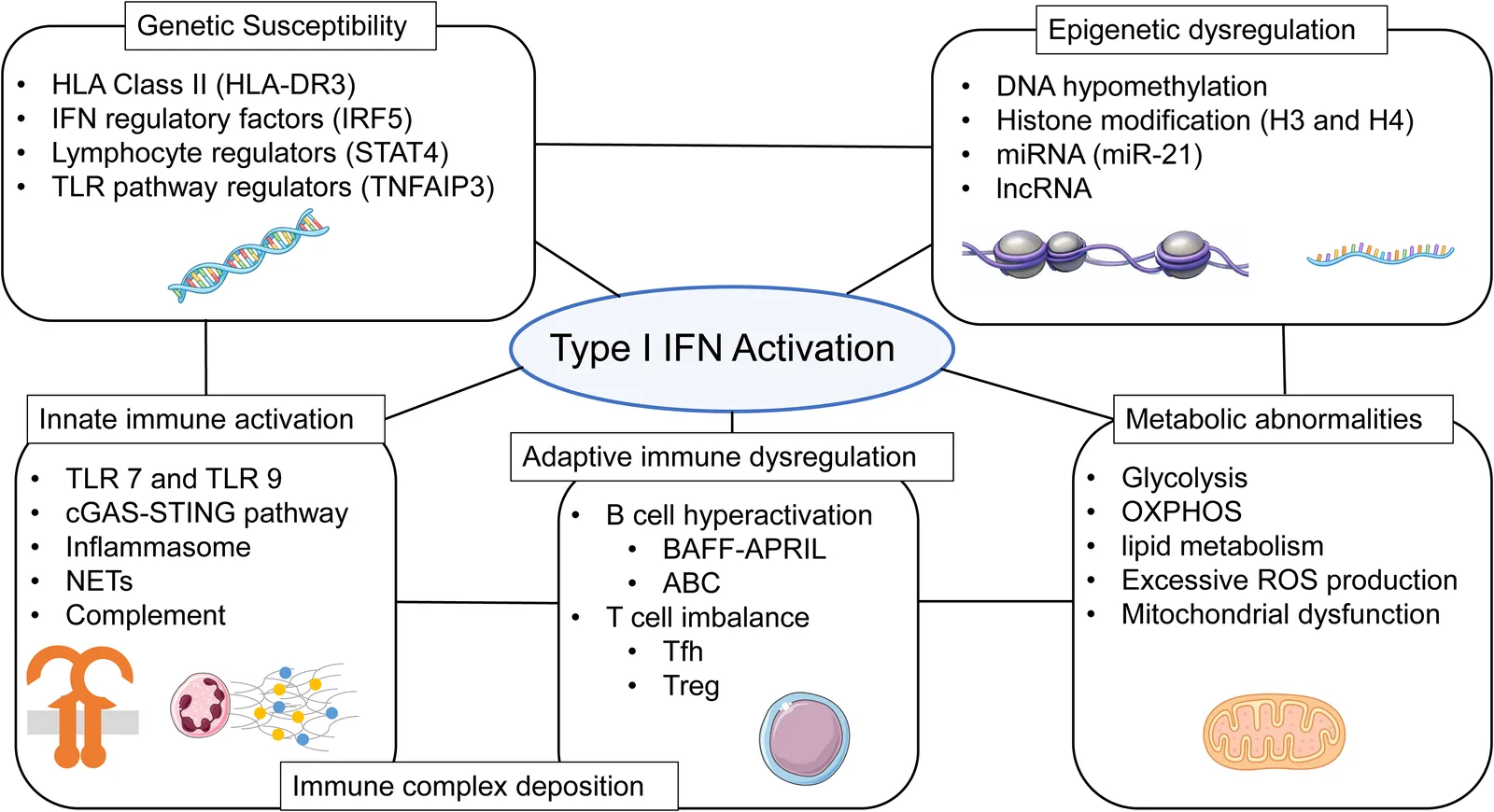
Molecular architecture of SLE pathogenesis. Integrated molecular architecture of SLE pathogenesis. Genetic susceptibility, epigenetic remodeling, innate nucleic acid sensing, B and T cell dysregulation, immune complex-mediated tissue injury, and immunometabolic abnormalities interact bidirectionally to generate heterogeneous molecular endotypes. Type I IFN is a major disease-associated hub. ABC, age-associated B cell; APRIL, a proliferation-inducing ligand; BAFF, B cell activating factor; cGAS, cyclic guanosine monophosphate-adenosine monophosphate synthase; DNA, deoxyribonucleic acid; HLA, human leukocyte antigen; IFN, interferon; lncRNA, long non-coding ribonucleic acid; miRNA, micro ribonucleic acid; NET, neutrophil extracellular trap; OXPHOS, oxidative phosphorylation; ROS, reactive oxygen species; SLE, systemic lupus erythematosus; STING, stimulator of interferon genes; Tfh, T follicular helper; TLR, Toll-like receptor; Treg, regulatory T cells. Adapted from Servier Medical Art (https://smart.servier.com), licensed under CC BY 4.0 (https://creativecommons.org/licenses/by/4.0/).

## 2 Conceptual and operational framework for molecular endotypes in SLE

### 2.1 Definition of molecular endotypes and a pragmatic four-program framework

In this review, the term “molecular endotype” is used to denote a biologically coherent and measurable disease state characterized by a reproducible constellation of molecular and cellular features that reflects a dominant pathogenic mechanism and is associated with clinically relevant phenotypes or outcomes. A single elevated pathway score, cytokine concentration, cell population, or biomarker does not, by itself, establish an endotype.

Therefore, the evidence is classified into three levels in this review. A pathway signature indicates activation of a molecular pathway or cellular program. A candidate molecular endotype is supported by replicated molecular–clinical associations across patients or cohorts. A clinically actionable endotype additionally requires prospective evidence that a predefined biomarker or molecular state identifies differential benefit from a specific therapy relative to an appropriate comparator. At present, SLE has several candidate molecular endotypes, but no universally accepted molecular taxonomy or routinely implemented endotype-guided treatment algorithm.

This review presents four programs: IFN/IFN-stimulated gene (ISG), B cells/plasmablasts, neutrophils/neutrophil extracellular traps (NETs), and immunometabolic programs. However, these are not proposed as definitive, exhaustive, or mutually exclusive classifications. Rather, they represent broad, partially overlapping biological axes that recur across published datasets and provide a practical framework for comparing molecular classifications that would otherwise be inconsistent. Among these programs, IFN/ISG-, B-cell/plasmablast-, and neutrophil/NET-dominant states have been repeatedly observed across transcriptomic, cellular, and functional studies. In contrast, the immunometabolic program is presented as an emerging mechanistic axis because reproducible patient-level classification, practical biomarker criteria, and prospective treatment-response validation remain limited. Complement and immune-complex activation are considered cross-cutting effector processes that can amplify tissue injury across several dominant programs rather than constituting a mutually exclusive fifth endotype.

### 2.2 Reconciliation with published molecular taxonomies

As mentioned above, various studies have reported different molecular groups. Different studies have reported three, five, six, or eight molecular groups because the number and composition of data-driven clusters depend on the assay platform, sample compartment, cohort characteristics, disease activity, treatment exposure, sampling time, feature selection, and clustering algorithm (8–11). These clusters should therefore not be mapped one-to-one onto the four programs proposed here. A single data-driven cluster may combine several programs, whereas one broad program may be divided into multiple clusters according to its intensity, cellular source, organ compartment, or accompanying molecular features. Our four-program framework should therefore be viewed as a low-dimensional, translationally oriented projection of higher-resolution molecular taxonomies rather than as an alternative clustering solution.

Endotype assignment should be regarded as both time- and compartment-dependent. Individual patients may express more than one molecular program simultaneously, and the relative dominance of each program may change with disease activity, flare, remission, infection, and treatment exposure. Endotype assignment should therefore specify the biological compartment and time point at which it was measured.

Blood-based signatures enable repeated and minimally invasive assessment of systemic immune activity, but they may not capture kidney-, skin-, or central nervous system-specific immune-stromal niches. Conversely, tissue-dominant programs may be weak or undetectable in peripheral blood. In lupus nephritis, blood profiling should therefore be integrated with urinary biomarkers, renal histopathology, and, where available, tissue transcriptomic or spatial information. Longitudinal paired sampling is required to distinguish relatively stable susceptibility axes from reversible activity states and treatment-induced molecular changes.

Ancestry-associated variation in molecular-program distributions has been reported, although the findings remain cohort- and assay-dependent. In adult ILLUMINATE datasets, Hubbard et al. identified differences in endotype frequencies across reported ancestry groups, including greater representation of a plasma-cell-enriched subgroup among patients of African ancestry, while most endotypes were shared across groups (9). Catalina et al. identified ancestry-associated differences in B cell/plasma-cell and myeloid signatures, with important contributions from autoantibody profiles and background treatment (12). Cell-sorted transcriptomics in Asian and White patients likewise identified ethnicity-associated molecular clusters (13). However, these observations should not be interpreted as ancestry-determined disease mechanisms: self-reported race/ethnicity and genetically inferred ancestry are not interchangeable, and blood-cell composition, disease activity, treatment exposure, and recruitment context can influence the observed distributions. These studies do not establish a harmonized ancestry-stratified distribution of the four programs used in this review or justify assigning an individual patient’s endotype or treatment on ancestry alone. Accordingly, population context should inform the interpretation and external validation of molecular profiles, rather than substitute for direct measurement. Future studies should evaluate program distributions and biomarker performance across well-characterized populations using standardized assays.

## 3 Genetic and epigenetic determinants of endotype states

### 3.1 Genetics

Research on the genetic associations of SLE began with the discovery of disease-associated human leukocyte antigen (HLA) in 1971, and strong heritability has been established through familial aggregation and twin studies (14–16). Subsequently, advances in genome-wide association studies (GWAS) have led to the discovery of over 300 estimated risk loci (17, 18). GWAS have revealed that HLA class II variation, interferon pathway genes, lymphocyte signaling regulators, and genes involved in nucleic-acid clearance shape polygenic susceptibility, whereas deficiencies in complement or nucleases can define monogenic or early onset lupus-like disease (17, 19–28). In contrast, research comparing East Asians and Europeans has observed that while the direction and magnitude of allele effects were comparable, Asians exhibited a higher frequency of risk alleles and a higher proportion of population-specific risks than Europeans, indicating racial differences in risk loci (29). Thus, the qualitative structure (the specific pathways and genes) is strongly shared globally, and quantitative differences arise from rare ancestor-specific variants and the extent to which each ancestral population carries more common risk alleles.

Several monogenic pathways associated with lupus-like diseases (monogenic lupus) have been reported (30, 31). Genetic defects in the classical pathway of complement are frequently associated with monogenic lupus, and numerous genes involved in nucleic acid metabolism and sensing, type I IFN and lymphocyte signaling regulators, and apoptosis are known to cause monogenic lupus (32–35). A very small proportion of patients with SLE possess highly penetrant mutations in a single gene that are sufficient to cause the disease. However, heterozygous rare deleterious variants in genes associated with monogenic SLE may also contribute to disease susceptibility in a subset of patients with childhood-onset SLE (36). In several SLE cohorts with childhood-onset disease, 13-25% of individuals carried rare variants predicted to be associated with SLE (37, 38). Sayadi et al. reported that in patients with childhood-onset SLE, the presence of a rare damaging single nucleotide variant, even in a heterozygous state, contributes to the disease process (36). Conceptually, SLE risk is thought to exist on a spectrum ranging from single-gene disorders or disorders involving a small number of genes (with a few variants having large effects, often associated with early onset) to typical polygenic disorders (involving many variants with small effects), with numerous individuals falling between these extremes.

### 3.2 Epigenetics

Abnormal epigenetic regulation is also strongly associated with the pathogenesis of SLE, and environmental factors are thought to play a regulatory role through epigenetic mechanisms and contribute to SLE onset. DNA methylation changes are among the most consistent epigenetic features associated with SLE. Global DNA hypomethylation is a hallmark of SLE, particularly pronounced in CD4^+^ T cells (39). The degree of hypomethylation correlates with disease activity, and in SLE CD4^+^ T cells, the following methylation-sensitive genes are overexpressed: cytokine genes (*IL4*, *IL6*, *IL10*, *IL13*), co-stimulatory molecule genes (*CD70*, *CD6*, *CD11A*, *CD40LG*), and ISG (*IFIT1*, *MX1*, *STAT1*, *TRIM22*, *BST2*) (40, 41). A multi-omics analysis revealed that the expression of ISG declines with aging, and this decline correlates with changes in genomic methylation patterns, suggesting that it may contribute to the improvement of SLE with age (42). Another study reported that the elevated expression of SETD3 in SLE CD4^+^ T cells may enhance methylation and acetylation in the Bcl-6 promoter region, potentially leading to an increased proportion of T follicular helper (Tfh) cells (43). Furthermore, DNA methylation in T cells, B cells, monocytes, and dendritic cells has been reported to be associated with SLE, suggesting that methylation in various cell types is implicated in the pathogenesis (44–46).

Post-translational modifications of histone proteins are also major epigenetic mechanisms. It has been confirmed that the overall acetylation of histones H3 and H4 is reduced, and the overall methylation of histone H3K9 is reduced (47, 48). Changes in histone acetylation and methylation have been observed in diverse cell types, including B cells, CD4^+^ T cells, Tfh cells, and Th17 cells (49–51). Integrating the assay for transposase-accessible chromatin using sequencing (ATAC-seq) and RNA-seq in CD4^+^ T cells identified five major transcription factors (ZNF770, THAP11, ZBTB14, ETV1, and POU3F1) predicted to bind to differentially accessible regions (DARs), and that DARs showing increased accessibility in lupus were enriched for lymphocyte activation, T cell activation, and cytokine signaling pathways (IFN-γ, tumor necrosis factor (TNF)-α, IFN-α) (52, 53). Another study employing ATAC-seq and single-cell RNA-seq on CD4^+^ T cells revealed that CCR7^low^CD74^hi^ regulatory T cells (Tregs) exhibit type I IFN-associated functional exhaustion, along with consistently abnormal expression patterns in both transcriptome and chromatin accessibility (54). In summary, histone modifications and chromatin accessibility signatures correlate with disease severity and create a permissive chromatin landscape that activates IFN, cytokine, and costimulatory genes while silencing tolerance-maintaining genes.

MicroRNAs (miRNAs) and long non-coding RNAs (lncRNAs) are important gene expression regulators in epigenetics. Previous studies have reported numerous miRNAs exhibiting abnormal expression levels in patients with SLE and lupus-model animals (55). However, there is little overlap in the miRNAs identified across studies, which may be due to the heterogeneity of SLE (1). A systematic review examining miRNAs in T cells in autoimmune diseases (with SLE being the most common) reported the increased expression of miR-21, miR-148a, and miR-155, as well as the decreased expression of miR-146a (56). There have been reports on other miRNAs, such as miR-146a, miR-200a-3p, and miR-203 (57–59). Regarding lncRNAs, there are reports indicating that lncRNA CASC2, lncRNA NEAT1, and lncRNA RP11-273G15.2 are associated with disease activity in SLE (60–63). A study integrating ATAC-seq and RNA-seq to analyze chromatin accessibility signatures in peripheral blood mononuclear cells (PBMCs) from patients with SLE showed IFN relationships of lncRNA–miRNA–genes and revealed a reduction in NK cell-mediated cytotoxicity pathways and recovery after treatment (64). Furthermore, recent advances in single-cell multi-omics technologies (e.g., scRNA-seq, scATAC-seq, Cellular Indexing of Transcriptomes and Epitopes by Sequencing [CITE-seq], and methylation profiling) have shown that each major immune compartment in SLE undergoes distinct epigenetic programs shaped by disease activity, genetic risk, and treatment (65). Elucidating cell type-specific epigenetics reflecting the functional differentiation of T cells, B cells, monocytes, and natural killer (NK) cells will be essential in the future. However, the diagnostic and mechanistic utility of these miRNAs and lncRNAs has not yet been established, and confirmation of reproducibility across multiple independent cohorts and cell types is required.

## 4 IFN/ISG-dominant candidate endotype program

### 4.1 Nucleic-acid sensing and IFN biology

In many patients with SLE, it has been reported that nucleic acid sensing pathways are chronically overactivated. These pathways include endosomal Toll-like receptors (TLR) 3/7/9, cyclic guanosine monophosphate-adenosine monophosphate synthase (cGAS) and its downstream the stimulator of IFN genes (STING), absent in melanoma 2 (AIM2)-like receptors (ALRs), and retinoic acid-inducible gene I (RIG-I)-like receptors (RLRs)- the mitochondrial antiviral signaling protein (MAVS) (1, 66). Cytosolic RNA is sensed by RLRs, such as RIG-I and melanoma differentiation-associated gene 5 (MDA5), which signal through MAVS, whereas cytosolic DNA activates cGAS, leading to cyclic GMP–AMP (cGAMP) production and STING–TANK-binding kinase 1(TBK1)–IFN regulatory factors (IRF) 3 signaling. The overview of the IFN pathway is shown in **Figure 2**.

**Figure 2:**
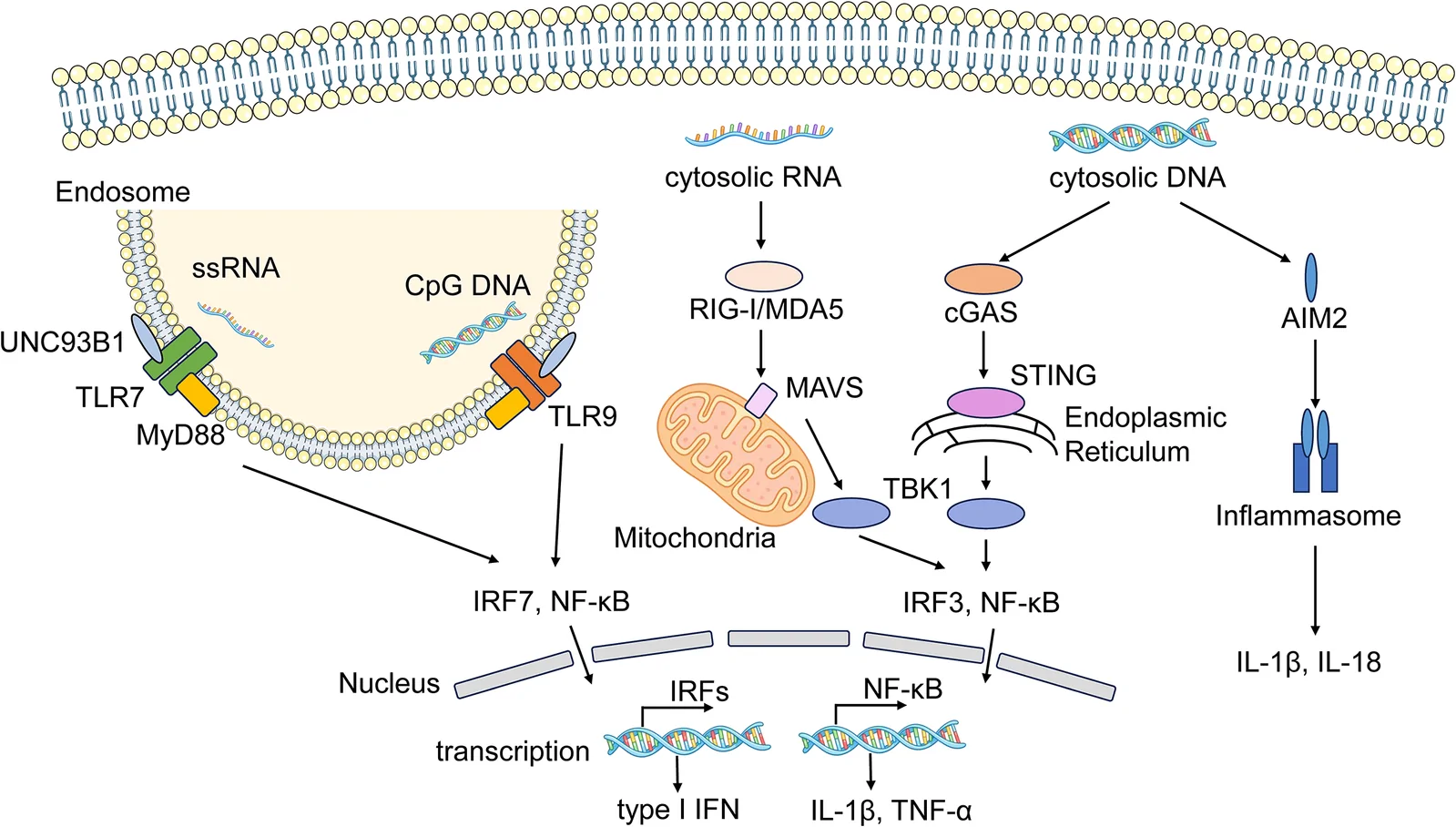
Overview of the IFN pathway in SLE. Key innate nucleic-acid sensing pathways relevant to SLE. Endosomal TLR7/TLR9 signaling, cytosolic DNA sensing via cGAS–STING, and cytosolic RNA sensing via RIG-I/MDA5–MAVS signaling converge on IRF/NF-κB activation and type I IFN production. AIM2 contributes to inflammasome activation and downstream IL-1β/IL-18 production. AIM2, absent in melanoma 2; cGAS, cyclic guanosine monophosphate-adenosine monophosphate synthase; CpG DNA, cytosine-phosphate-guanine deoxyribonucleic acid; DNA, deoxyribonucleic acid; IFN, interferon; IL, interleukin; IRF, interferon regulatory factors; MAVS, mitochondrial antiviral signaling protein; MDA5, melanoma differentiation-associated gene 5; MyD88, myeloid differentiation primary-response gene 88; NF-κB, nuclear factor kappa-light-chain-enhancer of activated B cells; RIG-I, retinoic acid-inducible gene I; RNA, ribonucleic acid; SLE, systemic lupus erythematosus; ssRNA, single-stranded ribonucleic acid; STING, stimulator of interferon genes; TBK1, TANK-binding kinase 1; TNF, tumor necrosis factor; TLR, Toll-like receptor. Adapted from Servier Medical Art (https://smart.servier.com), licensed under CC BY 4.0 (https://creativecommons.org/licenses/by/4.0/).

TLR3 recognizes double-stranded RNA, TLR7 recognizes single-stranded RNA, and TLR9 recognizes unmethylated cytosine-phosphate-guanine (CpG) DNA. TLRs are important in the pathogenesis of SLE. In humans, B cells and plasmacytoid dendritic cells (pDCs) express TLR7 and TLR9 within endosomes, where they recognize RNA and DNA delivered by immune complex Fc receptor-mediated uptake (67). Variants in UNC93B1, which is essential for TLR transport from the endoplasmic reticulum to endosomes and act as a scaffold for TLR dimerization, have been discovered in patients with early onset SLE (68). The report indicated that TLR7 stimulation in PBMCs from these patients resulted in increased production of TNF-α and interleukin (IL)-6. However, while an improvement in the disease was observed in TLR7-deficient lupus model mice, a worsening of the disease and increased serum levels of IgG and IFN-α were observed in TLR9-deficient lupus-model mice. TLR7 and TLR9 have opposing effects, which are thought to be mediated by differences in the intracellular signaling (TIR) domains of TLRs (69). It is also thought that the antagonistic effects of TLR7 and TLR9 may arise from factors such as the balance between the receptors and differences in the ligand environment.

IFNs are classified into types I (IFN-α, IFN-β, IFN-ω, IFN-κ, and IFN-ϵ), II (IFN-γ), and III (IFN-λ1, IFN-λ2, IFN-λ3, and IFN-λ4). In numerous SLE patients, elevated IFN concentrations are observed in the blood, and the overexpression of genes involved in type I IFN-mediated signaling is detected in PBMCs, a phenomenon termed the “IFN signature (1).” High levels of type I IFN activity are characteristic of SLE compared to other rheumatic diseases and are associated with the presence of cytokines such as B cell activating factor (BAFF) and type II IFN, autoantibodies such as anti-Ro52 antibodies and anti-ribonucleoprotein (RNP) antibodies, and clinical manifestations such as fever, hematologic disorders, mucocutaneous manifestations, and LN (70, 71). It is known that IFN-α activity is elevated in the serum of patients with SLE (72). In contrast, IFN-β production was increased in bone marrow-derived stem cells from SLE patients, and epidermal IFN-κ plays an important role in cutaneous lupus erythematosus (CLE) (73–75). These reports suggest that not only IFN-α, but also other type I IFNs are important. SLE risk variants are enriched in genes encoding components and regulators of the IFN alpha receptor (IFNAR) pathway, supporting the causal contribution of enhanced IFNAR–the Janus kinase (JAK)–signal transducer and activator of transcription (STAT) signaling to disease susceptibility and severity. In one bioinformatics study, IFIT1, RSAD2, ISG15, IFIT3, MX1, USP18, IFI44, OAS2, IFIT2, and OASL were identified as particularly important hub genes among ISGs, and reverse transcription quantitative polymerase chain reaction (RT-qPCR) showed that RSAD2, USP18, IFI44, and ISG15 were significantly upregulated in SLE patients compared to healthy controls (76). The expression levels of these ISGs correlate with laboratory test parameters and have been shown to exhibit high diagnostic performance in distinguishing SLE patients from healthy controls. The IFN signature, specifically changes in the ISG expression, links genetic risk factors to immune dysregulation, such as autoantibodies in SLE.

### 4.2 Transcriptomic ISG-high state versus functional IFN activity

The term “IFN/ISG-dominant program” is preferable to “type I IFN-dominant endotype,” because an elevated ISG score is not equivalent to demonstrably increased type I IFN bioactivity. Recently, it has been shown that high expression of ISG often diverges from directly measured levels of functionally active type I IFN proteins. Baechler et al. revealed that, although expression of ISG was elevated in PBMCs in half of SLE patients, there were no differences in serum IFN-α protein or messenger RNA (mRNA) levels (77). It was also shown that the ISG signature, once established, tends to be temporally stable and does not correlate with short-term fluctuations in disease activity (78, 79). Gómez-Bañuelos et al. demonstrated that four-gene IFN signature (IFI27, IFI44, IFI44L, and RSAD2) strongly correlates with the ISG module, but poorly correlates with type I IFN activity levels (80). The four-gene IFN signature showed high sensitivity (93%) but a low specificity (38%) in identifying SLE patients with high type I IFN activity. Moreover, systemic manifestations may be associated with combined type I, II, and III IFN activity rather than with an isolated type I IFN program.

### 4.3 Candidate biomarkers and therapeutic implications

Recent studies have also reported novel biomarkers as type-I IFN surrogate markers. In SLE, serum soluble SIGLEC-1 (CD169) levels are elevated and associated with renal complications and lower serum complement component 3, and are correlated with the ISG expression in monocytes (81, 82). The galectin-9 and CXCL10 are associated with type I IFN (83–86). Several studies have shown that MxA/Mx1 functions as a pathway biomarker for systemic type I IFN activation and that its role as an indicator of disease activity is limited (87, 88). In contrast, serum/urinary CD163 levels have been reported to serve as markers of LN and macrophage activity. Yang et al. reported that serum soluble CD163 levels correlate with LN severity (89). Soluble urinary CD163 also correlated with LN severity, and scRNA-seq revealed that M2 macrophages were responsible for the elevated soluble urinary CD163 (90–92). Furthermore, soluble urinary CD163 has been reported to be useful as a predictor of treatment response and flare in patients with LN (93, 94).

An IFN/ISG-dominant state should ideally be characterized using a composite profile that combines a transcriptomic ISG score with these candidate surrogate markers including SIGLEC-1, MxA, CXCL10, or galectin-9. These measurements should be interpreted together with clinical phenotype, sample compartment, disease activity, and treatment exposure. This distinction also has therapeutic implications. IFNAR1 blockade inhibits type I IFN signaling but does not directly block type II or type III IFN receptors. A high ISG score alone should therefore not be assumed to constitute a prospectively validated treatment-selection biomarker for anifrolumab. Future studies should test whether combining transcriptomic and functional IFN measurements improves treatment-by-biomarker discrimination.

## 5 B cell/plasmablast-dominant candidate endotype program

### 5.1 BAFF/APRIL and autoreactive B cell activation

BAFF, also known as B Lymphocyte Stimulator (BLyS), is a protein that maintains B cell survival, autoreactive B cell selection, class switch recombination, and the maintenance of long-lived plasma cells. It exists in both membrane-bound and soluble forms, both of which can activate B cells. BAFF acts as a survival and adaptogenic factor for B cells, maintaining homeostasis after the transitional phase of B cell development, whereas a proliferation-inducing ligand (APRIL) is thought to act at a later stage, regulating the function and survival of antigen-experienced B cells (95). Similar to BAFF, APRIL exists in two forms: membrane-bound and soluble. Both soluble BAFF and soluble APRIL can be released as trimers. BAFF and APRIL can form heteromers composed of two BAFF molecules and one APRIL molecule or one BAFF molecule and two APRIL molecules. BAFF and APRIL bind to transmembrane activator and CAML interactor (TACI) and B cell maturation antigen (BCMA), whereas BAFF additionally binds to the BAFF receptor (BAFFR). BAFF binds to BAFFR with the highest affinity. BCMA has a lower affinity for BAFF than for APRIL, whereas TACI exhibits equivalent affinity for both ligands (96). A heteromer composed of two BAFF molecules and one APRIL molecule can bind to BAFFR; however, a heteromer composed of one BAFF molecule and two APRIL molecules or an APRIL trimer cannot bind to BAFFR. B cells can express all three receptors, but their expression may change depending on the stage of maturation.

The importance of BAFF in SLE has been clearly established. Mice transgenic for BAFF exhibit an increase in mature B cells and develop SLE-like symptoms, such as anti-DNA antibodies and immunoglobulin deposition in the kidneys (97). Groom et al. reported the formation of a positive feedback loop wherein BAFF stimulation upregulates the expression of TLR7/9, and TLR7/9 activation upregulates the expression of TACI on B cells (98). Multiple studies have reported elevated BAFF levels in SLE and suggested a correlation with disease activity (99–101).

Bruton’s tyrosine kinase (BTK) is expressed in most hematopoietic lineages, except T cells, and plays a central signaling role in B cells, monocytes/macrophages, neutrophils, and pDCs, positioning it to coordinate both adaptive and innate components of lupus pathogenesis. BTK is a key proximal component of the BCR signaling pathway. Upon BCR activation, BTK is activated via SRC kinase and other kinases, phosphorylating substrates such as PLCγ2 and activating transcription factors, including nuclear factor kappa-light-chain-enhancer of activated B cells (NF-κB) and Erk (102). Studies using lupus model mice have reported that BTK inhibition improves autoantibody levels and clinical symptoms, such as nephritis, arthritis, and skin lesions (103–106).

### 5.2 ABC and Tfh/Tph–B cell crosstalk

Age-associated B cells (ABCs) are a B cell subset exhibiting the phenotype T-bet^+^, CD21^low^, CD11c^+^, and CD11b^+^ and overlap with double-negative 2 (DN2) B cells. ABC possesses diverse functions, including phagocytosis, antibody secretion, release of pro-inflammatory cytokines and chemokines, antigen presentation, and T helper (Th) cell activation. ABC cells are stimulated by pathogens, IL-21, IFN-γ signaling, and TLR7/9 signaling via myeloid differentiation primary-response gene 88 (MyD88), rapidly differentiating into antibody-secreting cells that secrete IgM and IgG (107). ABCs have been found to be associated with autoimmune diseases. The proportion of ABC in the peripheral blood of patients with SLE is higher than that in healthy controls, and increased ABC levels correlate with SLE activity (108). In patients with CLE, ABCs are recruited in response to IL-21 and TLR7/9 signals and produce localized antibodies, including IgG2a, IgG2b, and IgG3 (109). It is also suggested that it promotes Tfh differentiation via antigen presentation. Considering the diverse roles of ABCs, research is advancing to explore them as biomarkers for diagnosis and prognosis and as targets for therapeutic interventions. **Figure 3** summarizes the crosstalk between Tfh cells and B cells, and between ABC and T cells.

**Figure 3:**
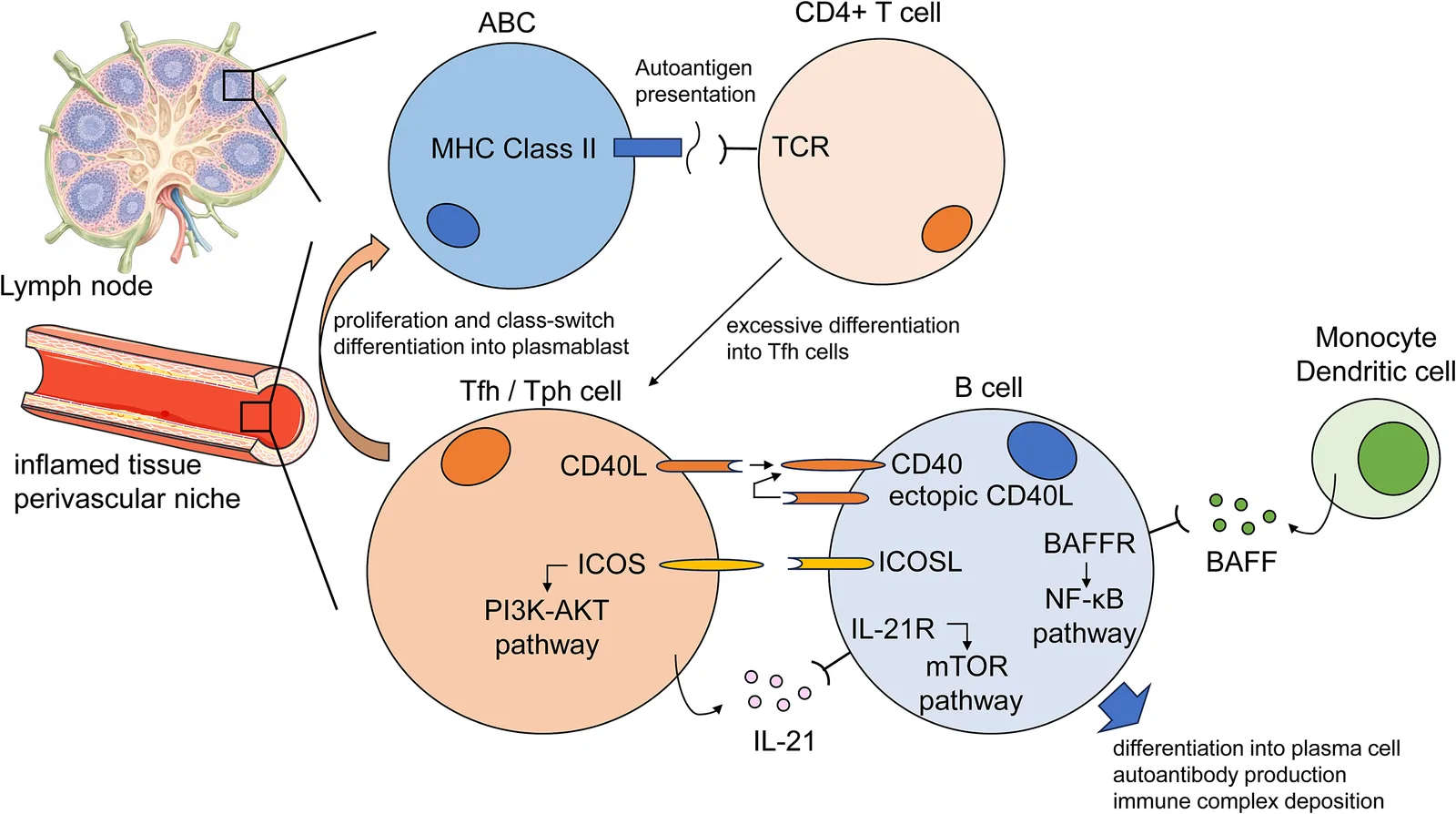
Crosstalk between Tfh/Tph and B cells, and between ABC and T cells. Adaptive immune network in SLE. Autoreactive B cells, including ABC-like populations, present antigens and receive help from Tfh/Tph cells via CD40–CD40L, ICOS–ICOSL, and IL-21 signaling. BAFF-rich myeloid niches promote survival, plasmablast differentiation, and autoantibody-mediated immune complex deposition. ABC, age-associated B cell; AKT, protein kinase B; BAFF, B cell activating factor; BAFFR, B cell activating factor receptor; CD, cluster of differentiation; ICOS, inducible T cell costimulatory; IL, interleukin; MHC, major histocompatibility complex; mTOR, mammalian target of rapamycin; NF-κB, nuclear factor kappa-light-chain-enhancer of activated B cells; PI3K, phosphoinositide 3-kinase; SLE, systemic lupus erythematosus; TCR, T cell receptor; Tfh, T follicular helper; Tph, T peripheral helper. Adapted from Servier Medical Art (https://smart.servier.com), licensed under CC BY 4.0 (https://creativecommons.org/licenses/by/4.0/).

Tfh and T follicular regulatory (Tfr) cell dysregulation is a hallmark of SLE, characterized by excessive Tfh activity and impaired Tfr suppression, leading to uncontrolled germinal center (GC) reactions and pathogenic autoantibody production in SLE. The mechanisms by which Tfr cells suppress immune responses include the inhibition of co-stimulatory signals via cytotoxic T lymphocyte antigen-4 (CTLA-4) and the secretion of cytokines such as IL-10 and transforming growth factor β (TGF-β). In patients with active SLE, circulating Tfh (cTfh) cells were significantly increased, whereas circulating Tfr (cTfr) cells were significantly decreased (110–112). It has also been reported that serum IL-21 levels correlate with these changes. IL-21, secreted predominantly by Tfh cells and their precursors, has pleiotropic effects that promote B cell proliferation, plasma cell differentiation, and antibody production. The Tfh/Tfr ratio is considered a more robust indicator of disease activity than the individual cell frequencies (111, 112). A recently proposed mechanism for this Tfh/Tfr imbalance involves the systemic circulation of pathogen-associated molecular patterns (PAMPs) due to intestinal barrier dysfunction (112).

Tfh cells and their peripheral counterparts (T peripheral helper [Tph] cells) provide multivalent signals to autoreactive B cells through coordinated CD40–CD40L engagement, IL-21 secretion, inducible T cell costimulator (ICOS) and its ligand (ICOSL) interactions, and cytokine-mediated support. In patients with SLE, circulating CXCR5^-^ PD-1^+^ Tph cells are increased (113, 114). Similar to Tfh cells, Tph cells can recruit and activate B cells through the production of CXCL13 and IL-21, driving B cell differentiation into plasmablasts or ABCs.

The CD40–CD40L interaction is a foundational signal in B cell–T cell cooperation and is pathologically dysregulated in patients with SLE. T cells express high levels of CD40L upon activation, whereas B cells express CD40. In patients with SLE, CD40L is overexpressed on T cell surfaces, and this increased expression correlates with disease activity (115). Additionally, the ectopic expression of CD40L on B cells has been observed in some patients with SLE (116). CD40L^+^ B cells can spontaneously produce antibodies, and transgenic mice expressing CD40L on B cells develop a lupus-like disease with age, suggesting their involvement in pathogenesis.

IL-21, mainly produced by Tfh cells, is also important for B cell–T cell crosstalk. IL-21 signaling in B cells synergistically interacts with the BCR and CD40 to activate the JAK–STAT and mammalian target of rapamycin (mTOR) cascades, leading to S6 phosphorylation and enhancing B cell response and proliferation (117). Furthermore, it has been reported that IL-21 maintains the expression of Bcl-6, a transcription factor essential for the GC response, in GC B cells (118). IL-21 can enhance the expression of MYC in GC B cells in cooperation with CD40; however, in lupus model mice, MYC is reduced, which is thought to lead to zonal recycling (119, 120).

The ICOS signaling pathway plays an essential role in the differentiation and function of Tfh cells. ICOS on Tfh cells interacts with ICOSL on B cells to activate the phosphoinositide 3-kinase (PI3K)–protein kinase B (AKT) pathway, thereby inducing Tfh cell localization within B cell follicles and inducing Bcl6 and c-Maf, transcription factors that positively regulate Tfh differentiation, while suppressing antagonistic transcription factors Blimp1 and T-bet (121). Khunsri et al. reported that in SLE patients, circulating Tfh1 cells (CXCR5^+^ PD-1^+^ CXCR3^+^ CCR6^-^) and circulating Tfh17 cells (CXCR5^+^ PD-1^+^ CXCR3^-^ CCR6^+^) exhibit the enhanced expression of ICOS relative to healthy controls, and that this expression correlates with disease activity (122). ICOS-ICOSL interaction is essential for GC formation, B cell–T cell interaction, and antibody production, whereas the inhibitory PD-1/PD-L1 axis serves as a homeostatic counterbalance, preventing excessive Tfh proliferation and antibody generation. The balance between these positive and negative signals likely determines whether B cell responses are controlled or become pathogenically amplified in SLE.

### 5.3 Short-lived plasmablasts versus long-lived plasma cells

A B cell/plasmablast-dominant state should not be regarded as a single uniform cellular compartment. At least two antibody-producing components should be distinguished: peripheral B cell/short-lived plasmablast activity and bone marrow long-lived plasma cell (LLPC) activity. Hoyer et al. demonstrated that approximately 40% of the antibody-producing cells in the spleens of lupus model mice were LLPCs; while cyclophosphamide treatment eliminated the short-lived plasmablasts, the LLPCs did not decrease in number and continued to produce antibodies (123). While LLPCs are the source of anti-RNP antibodies, short-lived plasmablasts are thought to be the primary source of anti-dsDNA antibodies (124, 125). It is suggested that type I IFN preferentially expands the population of short-lived plasmablasts, and chronic exposure to type I IFN in patients with SLE may continuously replenish this population of short-lived plasmablasts (126). Furthermore, in humans, LLPCs are found within the CD19^-^ CD138^+^ fraction of the bone marrow, and in patients with active SLE, there is a marked increase in LLPC-like cells in the peripheral blood (125). These cells have acquired the ability to autonomously secrete APRIL and IL-10, forming an autocrine survival signaling loop.

In recent years, obinutuzumab, which is anti-CD20 antibody, and anti-CD19 CAR T therapy have been tried as treatments for SLE (6, 127, 128). However, CD20-directed therapies primarily deplete mature B cells and do not directly eliminate CD20-negative plasmablasts or LLPCs. CD19-directed approaches cover a broader B-lineage compartment but may spare CD19-negative LLPCs, whereas BCMA-directed approaches are designed to target plasma-cell compartments (129–131).

### 5.4 Candidate biomarkers and therapeutic implications

Candidate readouts of peripheral B cell and plasmablast activity include the frequencies of ABCs and circulating plasmablasts, BAFF/APRIL concentrations, immunoglobulin transcriptional modules, and BCMA-related signals (131). Álvarez Gómez et al. revealed that in the peripheral blood of SLE patients, the frequency of CXCR5^-^ CD11c^+^ atypical B cell subsets, which include ABCs, is elevated, and that these cells are prone to differentiating into cells that secrete autoantibodies via the extrafollicular pathway (132). Since the number of circulating plasmablasts fluctuates in correlation with SLE disease activity, the plasmablast signature may serve as a biomarker reflecting disease activity (4). Furthermore, the plasmablast modules identified in gene expression analysis may be used as an axis to define the high-B cell endotypes (133). In contrast, persistent or recurrent pathogenic autoantibody production despite effective peripheral B cell depletion may suggest an important LLPC contribution, although a validated non-invasive biomarker of the LLPC compartment is currently lacking.

Distinguishing between these short-lived plasmablasts and LLPCs provide a mechanistic basis for considering BAFF blockade, anti-CD20 therapy, CD19-directed cellular therapy, BCMA-directed therapy, or dual CD19/BCMA targeting in different biological contexts. However, these biological readouts should currently be regarded as treatment-selection hypotheses rather than as a validated clinical algorithm.

## 6 Neutrophil/NET-Dominant candidate endotype program

### 6.1 NET formation and LDGs

NETs are key drivers of type I IFN production (134). NETs are formed as a defense mechanism by neutrophils, which physically trap microorganisms within structures containing DNA and antimicrobial peptides through a process called NETosis. NETs are rich in autoantigens in SLE, and NETosis has been reported to be associated with antinuclear antibodies, anti-dsDNA antibodies, and IFN signatures (134). IFI16, a member of the ALR family, binds to NETs in LN and AIM2, suggesting that both are NET-associated SLE autoantigens (135). In patients with SLE, impaired NET clearance occurs due to the presence of DNase1 inhibitors and anti-NET antibodies, and impaired NET clearance correlates with the titers of antinuclear antibodies and anti-dsDNA antibodies (136). Furthermore, NETs may be involved in the pathogenesis of SLE, including complement activation, induction of IFN-α synthesis, and tissue damage (137). NETs are rich in classical SLE autoantigens, such as dsDNA, citrullinated histones, myeloperoxidase, and neutrophil elastase (NE). NETs also contain the neutrophil antimicrobial peptide LL37 and human neutrophil peptides, which induce the production of autoantibodies against them, thereby activating pDCs (138). In contrast, in SLE patients, inadequate clearance occurs due to defects in DNase1/DNase1L3 activity, the inhibitory effect of anti-DNase antibodies, and shielding by NET-associated proteins such as AIM2 and IFI16 (135, 136, 139). These altered NETs provide key autoantigens and efficiently deliver nucleic acids to sensors (endosomal TLR7/9, cytosolic cGAS-STING, and inflammasome sensors such as AIM2/IFI16), thus sustaining type I IFN and IL-1/IL-18 production (140, 141). Immune complexes and autoantibodies trigger NETosis in SLE; therefore, reducing autoantibodies may lead to a reduction in NETosis. Combination therapy with rituximab and belimumab has been reported to reduce NETosis, and hydroxychloroquine has been shown to inhibit NET formation (142).

Low-density granulocytes (LDGs), frequently observed in patients with SLE, are defined as granulocytes that sediment into the PBMC fraction after density centrifugation. LDGs induce excessive NET production through increased mitochondrial reactive oxygen species (ROS) (143, 144). NETosis is largely driven by mitochondrial ROS rather than NADPH oxidase. Mitochondrial ROS alone can induce NETs enriched in oxidized mitochondrial DNA. The oxidized mitochondrial DNA derived from LDG NETs possesses potent pro-inflammatory activity and the ability to induce type I IFN production (144).

### 6.2 Oxidized mitochondrial DNA and NET-associated inflammation

Mitochondrial DNA (mtDNA), when oxidized and released through the formation of abnormal NETs, acts as a potent endogenous nucleic acid trigger that induces type I IFN production in SLE (144). In healthy neutrophils, oxidized mtDNA is selectively transported to lysosomes and degraded. However, in neutrophils from SLE patients, this degradation pathway is inhibited, causing the oxidized mtDNA to bind to the receptor for advanced glycation end-products (RAGE) receptor on the surface of pDCs, be internalized, and trigger the production of large amounts of type I IFN via TLR9 (145). Furthermore, Miao et al. revealed that oxidized mtDNA binds to active gasdermin D within the cell, forming pores in the cell membrane and inducing cell death (146). Through this mechanism, oxidized mtDNA is further released into the extracellular space, and inflammation is further exacerbated by a positive feedback loop. It was suggested that, compared to ANCA-associated vasculitis, oxidized mtDNA is more abundant in NETs in SLE, and the mitochondrial oxidative–NET–IFN axis may be characteristic of SLE (147).

### 6.3 Candidate biomarkers and clinical associations

Reported neutrophil-associated biomarkers in patients with SLE include LDG-phenotypes, transcriptomic modules, and soluble NET-derived markers. Denny et al. reported that LDGs in the PBMCs of SLE patients are activated and exhibit increased production of inflammatory cytokines such as TNF-α and IFN-γ, as well as type I IFN, and that patients with high circulating LDG frequency tend to have skin involvement and synovitis (148). LDGs are classified into two distinct subsets based on the presence or absence of CD10, and it was suggested that in SLE patients with higher levels of CD10^+^ LDG, which are more mature, these cells correlate with irreversible organ damage, impaired renal function, and cardiovascular complications (149). In recent years, while LDG is typically characterized by CD14^-^ and CD15^low^, a subset of LDG has been identified that exhibits LDG characteristics (CD14^+^, CD15^+^, CD16^+^, CD66^+^, HLA-DR^+^, and CD10^low^) while also expressing antigen-presenting cell markers (150). It was reported that this subset correlated with disease activity, inflammatory markers, and cardiovascular risk, and further detailed analysis is anticipated. Kegerreis et al. analyzed gene expression profiles obtained from isolated LDG from SLE patients, identified cell-type-specific gene modules, and reported their association with decreased levels of complement, anti-dsDNA antibody positivity, and the type I IFN signature (151). In another study, modular repertoire analyses were performed on microarray data, and a modular neutrophil signature is associated with LN (152). However, since these biomarkers, like other endotypes, have not yet been established, they require further validation across multiple cohorts.

## 7 Cross-cutting modifiers and effector axes

### 7.1 Complement/immune-complex-active effector axis

The complement system is a cascade of >30 plasma and membrane-bound proteins that serves three primary functions: tagging pathogens and damaged cells for destruction (opsonization), directly killing cells via membrane perforation (lysis), and recruiting inflammatory cells to sites of infection and tissue injury (inflammation). The removal of dead cells must be regulated to avoid excessive inflammation and autoimmune reactions, and complement plays an important role in this regulation.

Complement biology in SLE encompasses two conceptually distinct processes. Inherited deficiencies of early classical-pathway components impair the clearance of apoptotic material and immune complexes and thereby confer susceptibility to lupus. Deficiencies in C1q, C4, C1r/C1s, and C2 have been implicated in the pathogenesis of SLE (153–155). Proposed mechanisms include impaired clearance of immune complexes and apoptotic bodies, loss of self-mesenchymal cells, inappropriate tissue deposition, and impaired differentiation of T and B cells (156). In contrast, acquired activation of the pathways after disease onset can amplify inflammation and tissue injury independently of the initiating susceptibility mechanism. The alternative pathway is particularly relevant as an amplification loop that can enhance complement activation initiated by immune complexes, NETs, or damaged tissues. Deletions in H factor-related families, which are complement regulatory proteins in alternative pathway, were also reported to contribute to an increased risk of SLE onset (157). While a deficiency in mannose-binding lectin (MBL) in the lectin pathway has been associated with an increased risk of developing LN, it was reported that plasma MBL levels are elevated in patients with SLE, suggesting that maintaining appropriate levels is important for controlling inflammation (158, 159).

Low serum C3 and C4 remain among the most widely used molecular biomarkers in clinical SLE practice, but they incompletely capture real-time complement activation because their circulating concentrations reflect both consumption and synthesis (160). The utility of C4d levels bound to T cells, B cells, and erythrocytes (generally termed “cell-bound complement activation products”) has been suggested as a biomarker (161, 162). In recent years, it has been reported that the Multianalyte Assay Panel index, calculated using a logistic regression model that combines cell-bound complement activation products and autoantibodies, is useful as a diagnostic biomarker (163). Complement activation may coexist with IFN/ISG-, B-cell/plasmablast-, or neutrophil/NET-dominant programs (137, 164). At present, complement activation is better considered a clinically measurable cross-cutting effector axis than a fully validated, mutually exclusive SLE endotype.

### 7.2 Emerging immunometabolic program

Immune cells in patients with SLE exhibit metabolic abnormalities, leading to changes in their functional and inflammatory states. Recently, various analyses have been conducted from the perspective of immunometabolism within each immune cell population. Immune metabolism primarily depends on glycolysis and oxidative phosphorylation (OXPHOS) processes.

Both glycolysis and OXPHOS are enhanced in CD4^+^ T cells from SLE patients. Despite the enlarged and hyperpolarized mitochondria, ATP production is low, highlighting the prominence of enhanced glycolysis (165, 166). Glucose metabolism via the pentose phosphate pathway activates mTOR complex 1 (mTORC1), contributing to Th17/Treg imbalance. Furthermore, in CD4^+^ T cells, activation by CD3/28 stimulation induces glucose transporter 1 (Glut1)-dependent glycolytic reprogramming, shifting the balance between glycolysis and OXPHOS toward glycolysis (167, 168). In B cells from lupus model mice, glycolysis and OXPHOS are also enhanced (169). Chronic BAFF exposure increases the expression of glycolytic enzymes and glucose transporters, promotes glycolysis, enhances pyruvate influx into mitochondria, strengthens OXPHOS, and elevates mitochondrial membrane potential (170, 171). BAFF activates the PI3K–AKT–mTORC1 pathway, which increases mitochondrial mass and OXPHOS, supporting the survival and proliferation of autoreactive B cells. A multi-omics analysis in patients with SLE and healthy controls revealed associations between OXPHOS/mitochondrial dysfunction in transcripts, genes, and the epigenome, and type I IFN and OXPHOS signatures in memory B cells (172).

In renal macrophages, mTOR–hypoxia-inducible factor (HIF) 1α-dependent glycolysis is suggested to be an amplifying factor for antibody-mediated inflammation in LN. Research on LN has revealed that IgG binding to Fcγ receptor (FcγR) on macrophages in the kidney induces metabolic changes, leading to a switch to glycolysis (173). This metabolic reprogramming is required to generate proinflammatory mediators, including IL-1β, and is dependent on mTOR and HIF1α. The renal Hif1a, Il1b, and HK2 expression were upregulated and positively correlated, linking HIF1α-induced glycolysis to IL-1β-mediated kidney inflammation in the lupus model mice. A transcriptomic analysis of LN patient kidneys revealed a strong monocyte/macrophage gene signature in glomeruli that was positively correlated with glycolysis and inversely correlated with OXPHOS (174).

The importance of lipid metabolism has been highlighted in addition to glucose metabolism. In SLE CD4^+^ T cells, increased glycosphingolipid levels and altered raft profiles are observed within lipid rafts, which are critical cell membrane domains for regulating lymphocyte activation and function (175). Furthermore, CD38 is highly expressed in SLE T cells, and in CD4^+^ T cells, CD38 alters the lipid profile, affects calcium homeostasis, and suppresses IL-2 production (176, 177).

Metabolic abnormalities have been recognized as major causative factors in SLE, leading to the development of metabolic modulators. The pharmacological inhibition of glycolysis using 2-deoxyglucose (2DG), a non-metabolizable glucose analog, combined with metformin, improves disease in multiple lupus mouse models (165, 166). This mechanism operates through the suppression of pathogenic Tfh cells, which are glucose-dependent and sustain the germinal center response, driving pathogenic autoantibody production (178). Moreover, the therapeutic effect involves the gut microbiota (179). 2DG-induced metabolic reprogramming alters the composition and metabolites of the microbiome, and the transfer of microbiota from 2DG-treated mice may protect naïve mice from disease development.

As mentioned above, although immunometabolic abnormalities are prominent in SLE, current evidence does not establish a stable metabolic-inflammatory endotype equivalent to the IFN/ISG-, B cell/plasmablast-, or neutrophil/NET-dominant programs. These immunometabolic dysfunctions occur across multiple immune and tissue-resident cell types and may amplify other dominant inflammatory programs rather than define a discrete patient subgroup. A metabolism-enriched subgroup has been identified in transcriptomic taxonomy; however, practical biomarker criteria, reproducible clinical associations, and prospective treatment-response data remain limited (8). Therefore, this category is defined as an “emerging immunometabolic program” and is distinguished from a clinically validated therapeutic endotype. Longitudinal metabolomic and cellular bioenergetic studies linked to clinical outcomes and treatment-by-biomarker interactions will be required before this program can be used for patient selection.

### 7.3 Shared signaling and post-translational modifiers

Intrinsic T cell abnormalities remain important components of SLE pathogenesis beyond T cell help for B cells. T cell receptor (TCR) signaling dysfunction is a characteristic of SLE pathogenesis. Reduced expression of the TCR-associated CD3ζ chain and its substitution by the Fc receptor common γ chain (FcRγ) favor SYK recruitment and amplify TCR-induced calcium signaling, in contrast to conventional CD3ζ–ZAP-70 signaling (180). Serine/arginine-rich splicing factor 1 (SRSF1) contributes to promoting the expression of normal CD3ζ protein and is involved in maintaining the homeostasis of T cells, including Tregs (181–184). A genome-wide methylation screen followed by targeted validation revealed that, in contrast to the hypomethylation more typically reported in SLE, CD3Z hypermethylation was correlated with higher disease activity and more severe clinical manifestations, as well as with reduced CD3ζ expression in T cells (185). Reconstitution of CD3ζ in patient T cells can normalize proximal signaling and augment IL-2 production (186). Additionally, a meta-analysis revealed elevated Th17 cell and IL-17 levels in patients with SLE (187). In parallel, expanded double-negative T cells, defined as CD3^+^TCRαβ^+^CD4^-^CD8^-^ T cells, can produce IL-17 and infiltrate LN kidneys, complementing the Th17/IL-17 effector axis (188). Within the present framework, these signaling and effector abnormalities are considered mechanisms that may intersect multiple molecular programs rather than defining a mutually exclusive T cell endotype.

Shared signaling adaptors may modify the intensity of several molecular programs without defining a discrete patient subgroup by themselves. SH3 domain-binding protein 2 (3BP2), a cytoplasmic adapter protein, provides one example. 3BP2 binds to signaling molecules such as SYK and SRC family kinases, thereby activating downstream signaling (189–191). Experimental studies have demonstrated that tankyrase-mediated ADP-ribosylation of 3BP2 creates a recognition motif for the E3-ubiquitin ligase RNF146, resulting in ubiquitination and proteasome-dependent degradation of 3BP2, and that disruption of the 3BP2 degradation pathway induces inflammation and bone loss (192–195). It was reported that SRC/SYK-mediated tyrosine phosphorylation of TLR2 induced by 3BP2 induces cytokine production and inflammation (196–199). Deletion of 3BP2 ameliorates lupus-like phenotypes in murine models (200). Experimental lupus data support the idea that altered 3BP2 abundance and its post-translational regulation can modulate inflammatory set points; however, current evidence does not establish a distinct 3BP2-defined human SLE endotype. The tankyrase–3BP2–RNF146 axis is therefore best regarded as a cross-cutting post-translational regulator and a candidate therapeutic vulnerability requiring biomarker-based validation in patients, rather than as an independent molecular endotype.

## 8 Biomarker contexts and multi-compartment endotype validation

### 8.1 Activity, prognostic, pharmacodynamic and predictive biomarkers

To avoid conflating distinct biomarker applications, the candidate molecular readouts are classified according to their context of use. Activity biomarkers reflect the current biological or clinical state. Prognostic biomarkers identify the likelihood of a future clinical event, such as flare or organ damage, irrespective of a specific treatment. Pharmacodynamic or response biomarkers change after therapy and indicate biological target engagement or treatment-associated response. Predictive biomarkers identify differential benefits from a specific therapy relative to an appropriate comparator, ideally through a prospectively specified treatment-by-biomarker interaction. **Table 2** lists representative biomarkers classified according to this classification system. However, at present, many of the molecular markers available for SLE function more as signaling pathway, activity, prognostic, pharmacodynamic or response-associated biomarkers rather than as validated biomarkers for treatment selection. There are strong expectations for the future development of biomarkers for treatment selection. **Figure 4**; **Table 3** summarize a framework for candidate molecular endotype programs in SLE.

**Table 2:** Classified representative biomarkers.

Biomarker		Index	Reference
Activity biomarkers			
	urinary CD163	LN severity, active lesion in renal biopsy	(90–92)
	serum soluble CD163	LN severity	(89)
	galectin-9	SLEDAI-2K	(83)
	CXCL9	SLEDAI	(86)
	M2BPGi	SLEDAI-2K	(83)
	circulating plasmablast	SLEDAI	(4)
	APC-like LDG	SLEDAI, anti-dsDNA titer	(150)
Prognostic biomarkers			
	urinary soluble CD163	the risk of developing end-stage kidney disease	(93)
	serum CD163	Risk of renal function deterioration, assessed using a composite renal endpoint	(89)
Pharmacodynamic biomarkers			
	SIGLEC-1 on monocytes	reduced by glucocorticoid pulse therapy	(82)
	plasmablast signatures	decreased due to mycophenolate mofetil and intravenous cyclophosphamide	(4)
	neutrophil signatures	enhanced by the physiological effects of glucocorticoids	(4)
	Percentage of CD10^-^/CD10^+^ LDG Subset	Elevated due to glucocorticoid therapy	(149)
Predictive biomarkers			
	Unestablished		

APC, antigen presenting cell; CD, cluster of differentiation; CXCL, C-X-C motif chemokine ligand; dsDNA, double-stranded deoxyribonucleic acid; IFN, interferon; LDG, low-density granulocytes; LN, lupus nephritis; M2BPGi, mac-2-binding protein glycosylation isomer; SLEDAI, systemic lupus erythematosus disease activity index; SLEDAI-2K, systemic lupus erythematosus disease activity index 2000.

**Figure 4:**
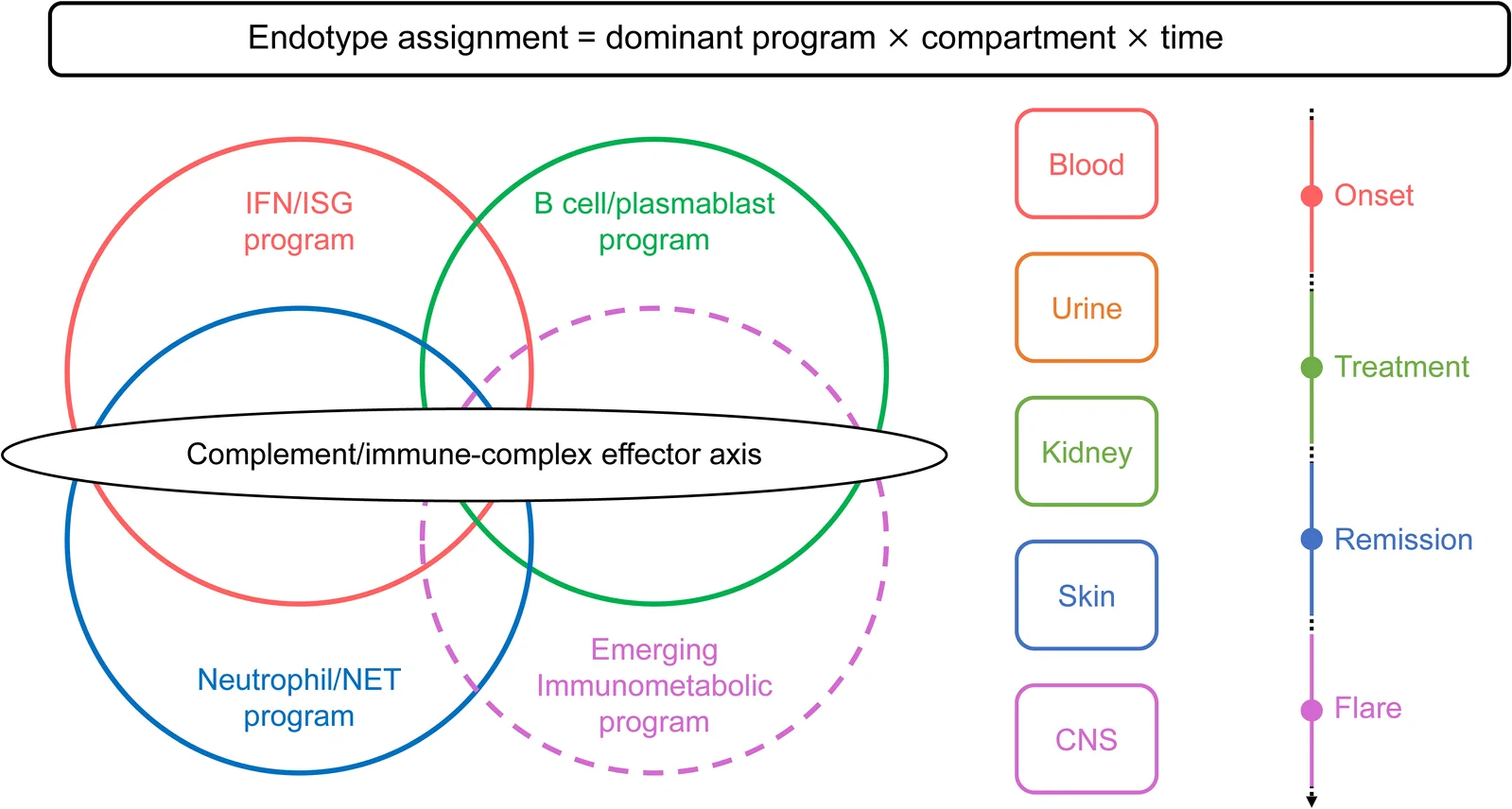
A pragmatic framework for candidate molecular endotype programs in SLE. Three recurrent dominant programs—IFN/ISG, B cell/plasmablast, and neutrophil/NET activity—are depicted as quantitative, overlapping, and non-mutually exclusive molecular states. The immunometabolic program is shown as an emerging program because evidence supporting stable patient-level classification and prospective treatment selection remains limited. Complement/immune-complex activation is depicted as a cross-cutting effector axis that may amplify tissue injury across several programs. Candidate biomarkers, reported clinical associations, and therapeutic evidence for these programs are summarized in **Table 3**. Individual patients may occupy different positions along these axes according to disease activity, treatment exposure, and organ compartment. Therefore, this framework should not be interpreted as a fixed one-patient/one-endotype classification. Abbreviation: IFN, interferon; ISG, interferon-stimulated genes; NET, neutrophil extracellular trap; SLE, systemic lupus erythematosus.

**Table 3:** Candidate endotype programs, biomarkers, therapeutic associations, and evidence gaps.

Program/axis	Defining features	Candidate biomarkers	Reported associations	Therapeutic target evidence	Predictive-selection evidence
IFN/ISG	ISG activation	ISG score, SIGLEC-1, MxA, CXCL10, and galectin-9	LN and systemic inflammatory manifestations	IFNAR1 target (anifrolumab) (214, 215, 262)	Prospective selection based on baseline ISG has not been established
B cell/plasmablast	BAFF/APRIL, ABCs, Tfh/Tph cells, and plasmablast/LLPC	The frequencies of ABCs and circulating plasmablasts, BAFF/APRIL concentrations, immunoglobulin transcriptional modules, and BCMA-related signals	Serologic activity, and autoantibody secretion	BAFF blockage (belimumab) and B cell depletion (obinutuzumab) (127, 211, 217–222)	Drug selection based on the B cell signature has not yet been established
Neutrophil/NET	LDG, NET formation, and oxidized mtDNA	Circulating LDG and CD10^+^ LDG frequency, and modular neutrophil signature	Skin involvement, synovitis, LN, and cardiovascular complications	Clinical trials of FcRn inhibition are currently underway (not a specific treatment for NETosis)	There is no standardized predictive assay yet
Emerging immunometabolism	Glycolysis, OXPHOS, mTOR–HIF1α pathway, and glycosphingolipid	Metabolomics, renal Hif1a, Il1b, and HK2 expression, and CD38 expression	Unconfirmed	Preclinical	Unconfirmed
Complement/immune-complex-active axis	classical/alternative activation, C3/C5 effector activity	C3/C4 levels, cell-bound complement activation products, and tissue deposition	LN, vasculopathic injury	Preclinical	Endotype-guided selection has not yet been established

ABC, age-associated B cell; APRIL, a proliferation-inducing ligand; BAFF, B cell activating factor; BCMA, B cell maturation antigen; CD, cluster of differentiation; CXCL, C-X-C motif chemokine ligand; FcRn, neonatal Fc receptor; HIF, hypoxia-inducible factor; HK2, hexokinase 2; IFN, interferon; IFNAR, interferon alpha receptor; ISG, interferon-stimulated genes; LDG, low-density granulocytes; LLPC, long-lived plasma cell; LN, lupus nephritis; mtDNA, mitochondrial deoxyribonucleic acid; mTOR, mammalian target of rapamycin; NET, neutrophil extracellular trap; OXPHOS, oxidative phosphorylation; Tfh, T follicular helper; Tph, T peripheral helper.

### 8.2 Single-cell analyses and spatial transcriptomics

Single-cell analyses and spatial transcriptomics have enabled the investigation of classification axes for SLE, defined by specific cells or lineages. In a study analyzing PBMCs from patients with SLE using scRNA-seq, the blood IFN signature arose from a limited set of ISG high subclusters within many lineages (ISG high CD14 monocytes, AXL^+^ SIGLEC6^+^ conventional dendritic cells (cDCs), pDCs, ABCs, ISG high plasma cells, ISG high T and NK clusters) (10). Hierarchical clustering of patients based on the frequency of these subclusters classified childhood SLE into six subcluster groups. The group with high disease activity exhibited a broad expansion of ISG high subclusters (ISG high monocytes, ABCs, and ISG high T and NK cells). Perez et al. reported that performing multiplexed scRNA-seq on PBMCs and classifying clusters based on the expression of individual module genes revealed associations with disease flares and anti-Smith antibody positivity (65). Molecular SLE subtypes have also been defined based on bulk blood transcriptomics. Cui et al. used blood microarrays to define three SLE subtypes: IFN-high, neutrophil/NE-high, and mixed, using a 92-gene signature (11). Hubbard et al. defined eight reproducible endotypes using transcriptomic profiling and machine learning (9). These findings were correlated with disease activity, autoantibody titers, complement levels, and lymphopenia, and suggested an association with treatment responsiveness. Moreover, Garantziotis et al. classified SLE into five molecular endotypes and examined the differences in drug responsiveness among them (8).

In addition to these systemic blood subtypes, organ-specific subtypes have been studied. A recent study involving healthy controls and SLE patients (active LN and non-nephritis controls) revealed that LN patients exhibited characteristic upregulation of specific TCR genes, such as TRBV27, TRBV15, and TRBV7-9, in CD8^+^ effector T cells, and activation of interactions mediated by macrophage migration inhibitory factor and its receptor complex (CD74 and CXCR4) (201). Additionally, this study demonstrated that an elevated κ/λ ratio in naïve B cells and an increased CD8^+^/CD4^+^ T cell ratio, and the model combining these factors, accurately identified LN patients (AUC = 0.895). scRNA-seq has also been performed on skin lesions, identifying subclusters such as CCL20^+^ keratinocytes, CXCL1^+^ fibroblasts, and GZMK^+^ CD8^+^ T cells, suggesting that keratinocytes and fibroblasts may also function as immune cells (202, 203). Spatial transcriptomics revealed that keratinocytes in discoid lupus erythematosus (DLE) exhibit enhanced type I IFN and complement pathways, with associated involvement of the RIG-I and cGAS–STING pathways (204, 205). Therefore, the classification of SLE is based on the systemic axes analyzed from single-cell blood and tissue axes, such as the kidney and skin, and it is thought that individual patients occupy different coordinates along these axes. However, at present, the definition of such subsets depends on each cohort, and since a unified classification has not yet been established, this remains a task for future research.

### 8.3 Multi-omics integration

Multi-omics research in SLE remains fragmented, but several examples exist in genomics, proteomics, metabolomics, and lipidomics. Huang et al. identified diagnostic biomarkers and therapeutic targets for SLE using a multi-omics analysis that integrated genomics, proteomics, and transcriptomics (206). In this study, they identified protein quantitative trait loci associated with genetic variants through a GWAS analysis and then investigated whether a causal relationship exists between plasma proteins and SLE susceptibility using Mendelian randomization. Next, they constructed a protein-protein interaction network using proteins suggested to be associated through a genomic analysis and identified three major genes and proteins (APOA2, MANSC1, and PSMB5) using machine learning. Finally, transcriptomic analyses, such as scRNA-seq, gene set variation analysis, and gene set enrichment analysis revealed that these genes are primarily expressed in T cells and monocytes and are associated with functions, including steroid biosynthesis, riboflavin metabolism, and DNA replication/repair mechanisms. Another study using genetic variation, protein, and metabolite data from the UK Biobank to predict the incidence and prevalence of SLE revealed that proteomic markers (five specified proteins) showed higher accuracy than metabolomic markers (five specified metabolites) and genomic markers (polygenic risk scores) (207). A multi-omics study analyzing blood samples from childhood SLE patients with varying disease activities integrated proteomics, metabolomics, and lipidomics (208). A proteomic analysis revealed an association between disease activity and the increased expression of apolipoproteins, such as apolipoprotein B (APOB). A lipidomic analysis showed increased sphingolipids, such as ceramides (Cer), and decreased glycerophospholipids, such as lysophosphatidylcholine (LPC), demonstrating a positive correlation between APOB and ceramides and a negative correlation between APOB and LPC. A metabolomic analysis revealed altered sphingolipid metabolism in patients during high-activity periods, with elevated levels of sphinganine and glucosylceramide and decreased levels of sulfatide and sphingomyelin. Combined with lipidomic analyses, sphingolipidomics is considered a useful tool for the early identification of SLE biomarkers. An integrated multi-omics analysis combining transcriptomics (mainly RNA-seq) and nuclear magnetic resonance (NMR)-based metabolomics in SLE neutrophils revealed that IFN-α/β signaling, neutrophil degranulation, and NET signaling were upregulated (209). Although multi-omics integration in SLE has been increasingly pursued in recent years, truly specialized tri-omics approaches (integrating genome, proteome, and metabolome data from the same patient) remain uncommon. In addition, more advanced frameworks (e.g., Multi-Omics Factor Analysis v2 [MOFA+], data integration analysis for biomarker discovery using latent components [DIABLO], and Bayesian multi-omics factor models) have yet to be widely applied. Although these multi-omics analyses are not yet mature enough for clinical implementation, they are useful for formulating hypotheses (such as those regarding endotypes), and further research is encouraged.

## 9 Therapeutic implications: from target validation to predictive selection

### 9.1 Established treatments with target validation

The term “established treatments with target validation” refers to those based on randomized or confirmatory clinical trials. Glucocorticoids, antimalarials, and conventional immunosuppressive agents remain the foundational treatment. In particular, mycophenolate mofetil and cyclophosphamide are considered important in the management of LN (210–213). In addition, anifrolumab, a human monoclonal antibody that binds to IFNAR1, demonstrated efficacy in SLE in phase IIb trial (MUSE) and phase III trial (TULIP-2) (214, 215). It has been suggested that anifrolumab may be effective in refractory CLE (216).

Belimumab is a monoclonal antibody that binds to soluble BAFF/BLyS and inhibits their binding to BAFFR, TACI, and BCMA on B cells. The efficacy of belimumab has been demonstrated in multiple phase III trials, and it is also available for subcutaneous administration (217–220). Furthermore, belimumab has shown renal efficacy in patients with active LN (221). An RNA-seq analysis of patients with active SLE who received belimumab for 6 months revealed that ISGs, such as IFIT1, IFI27, and IFI44, were upregulated in patients who responded to belimumab prior to treatment initiation (222).

Obinutuzumab (type II anti-CD20 antibody) has demonstrated efficacy against LN and SLE and is an approved treatment for active LN (127, 128). Relative to rituximab (type I anti-CD20 antibody), obinutuzumab exhibits enhanced antibody-dependent cellular cytotoxicity (ADCC), reduced CD20 internalization, and superior direct killing effects on B cells. The United States Food and Drug Administration (FDA) is currently reviewing obinutuzumab for approval for the treatment of SLE.

### 9.2 Clinical investigational therapies

Many targeted therapies for SLE are currently undergoing clinical trials. The following are some examples. Ianalumab is a fully human monoclonal antibody that targets BAFFR. It enhances binding to FcγR through afucosylation, thereby exerting dual effects, including B cell depletion via ADCC and BAFFR inhibition. A phase II randomized, double-blind, placebo-controlled trial of ianalumab was recently conducted, and a reduction in disease activity was observed with the combination of ianalumab and standard therapy (223). Mechanistically, ianalumab occupies an intermediate position between belimumab, which targets regulatory mechanisms and ligands, and obinutuzumab, which is a non-BAFF-specific depletor. Future research is warranted.

It has been reported that several BTK inhibitors improve nephritis and skin lesions in lupus model mice (103, 106, 224). Despite these results, clinical trial of BTK inhibitor for SLE showed limited efficacy (225). A new study on BAFFR-targeted BTK inhibitor nanocarriers has demonstrated improved disease control, and reduced levels of anti-dsDNA antibodies in lupus model mice; however, the research remains in the preclinical stage currently (226).

The neonatal Fc receptor (FcRn) normally binds circulating IgG in the endosome and rescues it from lysosomal degradation, extending IgG half-life (227). The monoclonal antibodies that block the IgG-binding site on FcRn accelerate degradation of both pathogenic and non-pathogenic IgG without depleting albumin, IgA, IgM, or IgE. In SLE, reducing total circulating IgG levels through FcRn inhibition is proposed an approach that complements therapies targeting B cells and cytokines. There is a report that FcRn inhibition in colitis reduces NETosis, suggesting that a similar mechanism may be involved in SLE as well (228). A Phase III clinical trial is currently underway for nipocalimab, a fully human anti-FcRn IgG1 monoclonal antibody.

Therapies targeting the JAK-STAT pathway have also been explored. Baricitinib is an ATP-competitive inhibitor of JAK1 and JAK2 that blocks signaling downstream of the type I IFN receptor. In a phase II trial, combination therapy with baricitinib (4 mg) and standard treatment demonstrated an improvement in disease activity (229). However, one of two subsequent phase III trials failed to meet the primary endpoint of an SRI-4 response at week 52, and a pooled analysis of both trials did not establish efficacy (230–232). Nevertheless, a transcriptomic analysis has suggested the existence of a subset of SLE that responds to baricitinib, and its efficacy needs to be re-evaluated (233). Deucravacitinib, a highly selective oral TYK2 inhibitor, met the primary endpoint of an SRI response (4) at week 32 in a phase II trial (234). Deucravacitinib has also shown efficacy against CLE, and results from an ongoing phase III trial are anticipated (235).

The CAR T cell therapy, previously used in cancer treatment, has been applied to SLE (6). T cells expressing a vector construct encoding CAR that binds to a specific antigen on target cells are used to eliminate B cells. The primary sources of autoantibodies in SLE are peripheral B cells (CD19^+^) and long-lived plasma cells in the bone marrow. CD19-directed approaches may spare CD19-negative LLPCs, providing a rationale for investigating BCMA-directed or dual CD19/BCMA-targeted strategies (129, 130). Research using allogeneic CD19-CAR T cells has also been conducted to overcome manufacturing delays and the cost of autologous products, suggesting their efficacy and safety (236). However, there is a possibility of adverse events such as immune effector cell-associated hemophagocytic lymphohistiocytosis-like syndrome (IEC-HS) and immune effector cell-associated neurotoxicity syndrome (ICANS), thus safety assessment is also necessary. The treatments described above are summarized in **Figure 5**.

**Figure 5:**
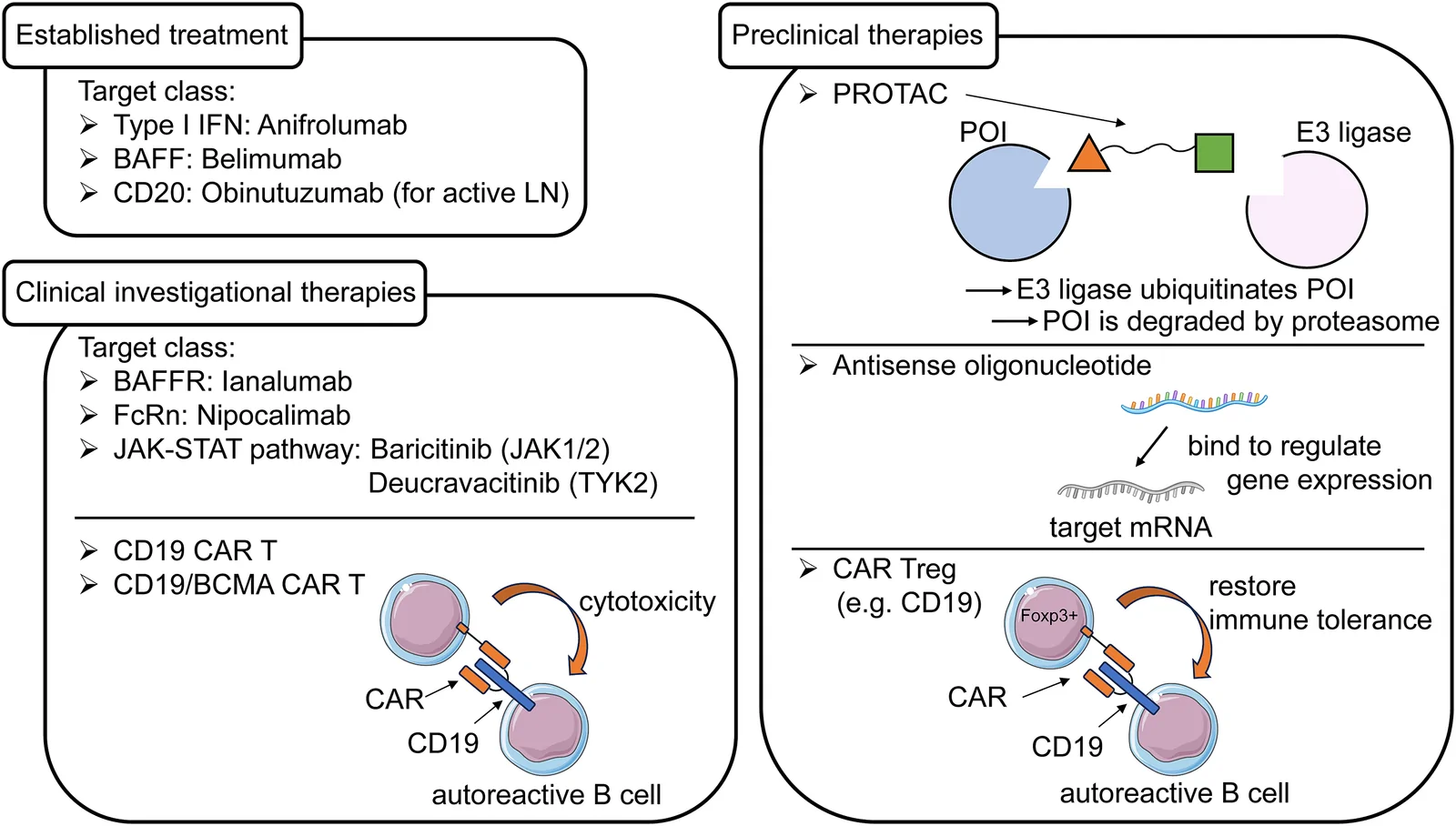
Established, investigational, and preclinical targeted therapies in SLE. Current and emerging molecular therapies for SLE. Established treatments include type I IFN inhibition, BAFF inhibition, and anti-CD20 therapy. Clinical investigational therapies include BAFFR inhibition, FcRn inhibition, JAK–STAT pathway suppression, and CAR T therapy targeting CD19 or CD19/BCMA. PROTAC, antisense oligonucleotides, and CAR Treg therapy are still in the preclinical stage, but further research is anticipated. BAFF, B cell activating factor; BAFFR, B cell activating factor receptor; BCMA, B cell maturation antigen; CAR, chimeric antigen receptor; CD, cluster of differentiation; FcRn, neonatal Fc receptor; IFN, interferon; JAK, Janus kinase; LN, lupus nephritis; MHC, major histocompatibility complex; mRNA, messenger ribonucleic acid; POI, protein of interest; PROTAC, proteolysis-targeting chimera; SLE, systemic lupus erythematosus; STAT, signal transducer and activator of transcription; Treg, regulatory T cells; TYK, tyrosine kinase. Adapted from Servier Medical Art (https://smart.servier.com), licensed under CC BY 4.0 (https://creativecommons.org/licenses/by/4.0/).

### 9.3 Preclinical or hypothesis-generating therapies

Recently, novel therapeutic strategies have been developed. First, proteolysis-targeting chimeras (PROTACs) are emerging as next-generation small-molecule modalities for SLE. PROTACs are bifunctional small molecules that bind a protein of interest (POI) with one ligand and an E3-ligase with the other, allowing the formation of a ternary POI–PROTAC–E3 complex (237). This complex drives the polyubiquitination of the POI and its subsequent destruction by the 26S proteasome; therefore, PROTAC acts catalytically. By repeating this process, the same molecule can repeatedly recruit new POI molecules to the E3 ligase. A BTK-targeting PROTAC demonstrated favorable results in a lupus mouse model (238). However, clinical trials targeting human SLE have not yet been conducted. The data suggesting that PROTAC may be beneficial are consistent with the broader view that PAR modification and ubiquitin-dependent regulation of signaling proteins may have therapeutic applications in immune-mediated diseases (197, 198). Second, antisense oligonucleotides (ASOs) are small nucleic acid sequences of approximately 15–25 nucleotides that hybridize with complementary RNA through base-pairing interactions (239). ASOs bind to target mRNA or precursor mRNA and regulate gene expression levels through mechanisms such as degradation and splicing. While it has been clinically applied in humans for neurological diseases such as spinal muscular atrophy, trials for lupus remain limited to research using mice (240, 241). Third, the efficacy of CAR Treg, human Tregs engineered to overexpress FoxP3 and express anti-CD19 CAR, has been suggested, and other autoantigen-specific TCR-engineered Treg therapies are being explored (242–244).

## 10 Future directions and challenges

In the future, personalized medicine based on molecular subtypes is expected to become crucial for SLE treatment. As mentioned earlier, genomics and transcriptomics have suggested the potential to classify SLE based on molecular subtypes (9, 11). However, such subtypes may change with disease activity or treatment and vary according to ethnicity (65). Attempts have also been made to analyze cell trajectories, examining how originally static single cells change during dynamic state transitions, such as flare, remission, organ involvement, and treatment (65, 202, 245, 246). For a unified classification, the timing and methods of classification must be carefully considered. Furthermore, multi-omics integration and artificial intelligence (AI)-based analyses are recognized as important tools for the diagnosis of SLE and stratification for treatment selection (208, 247–249). Future directions include graph-based models that encode known pathways and cell-cell interaction networks, transfer learning from larger population-based cohorts, and federated learning across institutions to mitigate privacy constraints while enlarging training data. However, challenges include the tendency of AI to frequently point to known pathways, such as IFN, BAFF/B cells, and oxidative stress; the small cohort size and heterogeneous treatments received; and the difficulty in capturing temporal changes due to the scarcity of longitudinal analyses. Therefore, future studies should focus on the integration of mechanism-based multi-omics signature development, AI methods capable of processing high-dimensional, longitudinal, and multimodal data, and prospective randomized trials evaluating the therapies predicted by these approaches.

Spatial omics technologies have rapidly advanced in molecular biology in recent years, enabling the acquisition of high-content omics data within tissues while preserving spatial localization information. Research has been conducted on LN, CLE, and NPSLE, investigating their cell dynamics and therapeutic effects. Tang et al. performed an integrated analysis of scRNA-seq and spatial transcriptomics using renal biopsy samples from patients with LN, mapping the cell types associated with LN (250). Single-cell-resolution spatial transcriptomics mapping *in situ* of childhood-onset LN revealed clusters of B cells and other cells forming tubulointerstitial immune hotspots (251). Steele et al. constructed a spatially resolved atlas of skin fibroblasts from spatial transcriptomics data of various skin diseases, including DLE, and analyzed the anatomical niches of fibroblasts (252). Aw et al. used a mouse model of NPSLE to perform multiplexed spatial transcriptomics via multiplexed error-robust fluorescence *in situ* hybridization (MERFISH), reporting that distinct ISG-positive patches exist in subcortical regions and that astrocytes and oligodendrocytes are involved (253). These spatial data analyses consistently suggest that organ damage in SLE is not driven by homogeneous inflammation but is rather organized around distinct immune-stromal niches. However, SLE spatial mapping is still under development, and it is desirable to construct an integrated multi-organ atlas covering various organs and match it with clinical data, such as peripheral blood and urine. In contrast, tissue sampling from organs such as the kidneys and central nervous system is highly invasive, limiting longitudinal studies in humans. Therefore, basic research using animal models will likely remain desirable.

Although epitranscriptome analyses are important, research on SLE remains insufficient. It has been suggested that RNA modifications, such as m6A, play an important role in SLE (254, 255). In addition, it has been reported that METTL3, the writer of m6A, is upregulated in peripheral B cells, but its role in specific subsets, such as Tfh and ABC, remains unclear (256, 257). There are few studies on other modifications, such as m5C and m1A, leaving the overall picture of RNA modifications unclear. However, elucidating the role of extracellular vesicles (EVs) is also important. Recent studies suggest that lncRNAs in exosomes may be involved in the activation of T cells and B cells and could serve as biomarkers for LN (258–261). However, research on EVs is limited. By combining future studies with the epitranscriptome, it will be possible to elucidate processes such as post-transcriptional RNA regulation and vesicle-mediated RNA transport.

## 11 Conclusion

SLE is now framed as a systems disease in which multi-layered molecular networks, including immune signaling, cellular metabolism, epigenetic and epitranscriptomic control, and dysregulated cell death/clearance, fail in concert rather than in isolation. High-dimensional omics have begun to map these layers and are driving a shift toward molecularly targeted therapies and data-driven molecular endotypes as the basis for precision medicine. Molecular endotyping in SLE should currently be viewed as a research framework toward precision therapy rather than as a treatment algorithm for routine clinical practice. Published studies have repeatedly identified IFN/ISG-, B-cell/plasmablast-, and neutrophil/NET-associated molecular states, whereas the immunometabolic program remains less clinically validated and may function primarily as a cross-cutting modifier. Complement and immune-complex activation similarly represent measurable effector processes that may overlap with several dominant programs.

Among the candidate programs, the IFN axis has the strongest clinical target-validation and pharmacodynamic evidence. However, no baseline molecular signature has yet been established as a universally applicable, prospectively validated treatment-selection biomarker. Evidence supporting B-cell-, neutrophil-, metabolic-, or complement-based therapeutic selection remains exploratory or preclinical to varying degrees.

Future progress will require standardized assays, longitudinal and multi-compartment sampling, external validation across diverse populations, and prospective trials that directly test biomarker-by-treatment interactions. Such studies will determine whether candidate molecular programs can be converted into reproducible and clinically actionable endotypes.
